# Genome-Wide SNP Identification and Characterization in Two Soybean Cultivars with Contrasting *Mungbean Yellow Mosaic India Virus* Disease Resistance Traits

**DOI:** 10.1371/journal.pone.0123897

**Published:** 2015-04-13

**Authors:** Chandra Bhan Yadav, Priyanka Bhareti, Mehanathan Muthamilarasan, Minakshi Mukherjee, Yusuf Khan, Pushpendra Rathi, Manoj Prasad

**Affiliations:** 1 National Institute of Plant Genome Research, New Delhi, India; 2 Department of Genetics and Plant Breeding, Govind Ballabh Pant University of Agriculture and Technology, Pantnagar, Uttarakhand, India; Louisiana State University Agricultural Center, UNITED STATES

## Abstract

*Mungbean yellow mosaic India virus* (MYMIV) is a bipartite Geminivirus, which causes severe yield loss in soybean (*Glycine max*). Considering this, the present study was conducted to develop large-scale genome-wide single nucleotide polymorphism (SNP) markers and identify potential markers linked with known disease resistance loci for their effective use in genomics-assisted breeding to impart durable MYMIV tolerance. The whole-genome re-sequencing of MYMIV resistant cultivar ‘UPSM-534’ and susceptible Indian cultivar ‘JS-335’ was performed to identify high-quality SNPs and InDels (insertion and deletions). Approximately 234 and 255 million of 100-bp paired-end reads were generated from UPSM-534 and JS-335, respectively, which provided ~98% coverage of reference soybean genome. A total of 3083987 SNPs (1559556 in UPSM-534 and 1524431 in JS-335) and 562858 InDels (281958 in UPSM-534 and 280900 in JS-335) were identified. Of these, 1514 SNPs were found to be present in 564 candidate disease resistance genes. Among these, 829 non-synonymous and 671 synonymous SNPs were detected in 266 and 286 defence-related genes, respectively. Noteworthy, a non-synonymous SNP (in chromosome 18, named 18-1861613) at the 149^th^ base-pair of *LEUCINE-RICH REPEAT RECEPTOR-LIKE PROTEIN KINASE* gene responsible for a G/C transversion [proline (CCC) to alanine(GCC)] was identified and validated in a set of 12 soybean cultivars. Taken together, the present study generated a large-scale genomic resource such as, SNPs and InDels at a genome-wide scale that will facilitate the dissection of various complex traits through construction of high-density linkage maps and fine mapping. In the present scenario, these markers can be effectively used to design high-density SNP arrays for their large-scale validation and high-throughput genotyping in diverse natural and mapping populations, which could accelerate genomics-assisted MYMIV disease resistance breeding in soybean.

## Introduction

*Mungbean Yellow Mosaic Virus* is one of the most destructive as well as widely distributed plant pathogenic viruses belonging to the family Geminiviridae. This virus causes yellow mosaic disease in legumes and particularly in India, the yields of several legume crops including blackgram (*Vigna mungo*), mungbean (*Vigna radiata*), cowpea (*Vigna unguiculata*) and soybean (*Glycine max*) are challenged by *Mungbean Yellow Mosaic India Virus* (MYMIV). Among legumes, soybean is an economically important crop in which MYMIV causes devastating yield losses [1–3]. MYMIV is transmitted by whitefly (*Bemisia tabaci*) and possesses bipartite, single stranded, circular DNA genome referred as DNA A and DNA B [4]. Both the genomes are of 2.5–2.7 kb in size and encode necessary components for replication, movement and symptom development [5,6]. Since 1970s, MYMIV is posing a major threat to Indian soybean cultivation and it is reported to spread throughout India in alarming proportions [7]. Therefore, it is imperative to generate elite soybean varieties with durable tolerance to MYMIV, either through molecular breeding or transgene-based approaches.

MYMIV is not sap-transmissible and transmission of these viruses by DNA abrasion was not reported. Thus, screening of host population depends on whitefly transmission, which varies with efficacy of transmission, vector biotype, growth conditions of the host, host-vector relationship and environment [8]. Although dissecting-out the genes that confer resistance to MYMIV is the proven way for controlling this disease, identifying the source of genetic resistance is a challenging task. In this scenario, wild-type soybean accessions could be a potential source of resistance, but high linkage drag along with partial sterility hinders the use of these wild accessions in breeding programmes. Due to complex genome architecture of soybean, there will be high interactions among the locus controlling different traits (agronomic and disease resistance), where linkage drag has been reported [9–11]. Therefore, a large-scale screening is necessary to identify stable source of resistance among the cultivated genotypes to facilitate the breeding for MYMIV resistance.

Deciphering the molecular genetic regulation of MYMIV disease resistance and manipulating the factors associated with the disease resistance machinery is the only effective strategy to control the yield loss in soybean. Construction of high-density genetic linkage map using large-scale co-dominant SNP markers is essential for fine mapping of genes/QTLs associated with MYMIV disease resistance in soybean. Therefore, the present study was conducted to; (i) identify a large number of SNPs and InDels showing polymorphism between MYMIV resistant soybean cultivar (UPSM-534) and susceptible Indian cultivar (JS-335), and (ii) screen the presence of these sequence variations in known disease resistant quantitative trait loci (QTLs). The information generated in this study will help to construct high-density genetic linkage-map for fine mapping and map-based cloning of genes/QTLs controlling MYMIV disease resistance in soybean.

## Materials and Methods

### Plant materials and disease evaluation

Seeds of MYMIV resistant soybean cultivar (UPSM-534; germplasm line derived from Taiwan, accession number ‘PI171443’) and highly susceptible Indian cultivar (JS-335; derived from JS 78–77 x JS 71–05) along with 10 other Indian soybean cultivars (GBPUA&T 1 to 10) were sown in pots (6 inch diameter) filled with vermiculite and agropeat, and grown in greenhouse at 26°C (15 plants from each cultivar in three replicates). At two-leaf stage, the seedlings were infected with *Agrobacterium* harbouring tandem dimers of both DNA A and DNA B of MYMIV following the protocol reported by Yadav et al. [8]. Percentage infectivity at 30 days post-inoculation (dpi) and initiation of symptom appearance were selected as criteria for scoring. Soybean cultivars were subsequently classified as resistant (R) and susceptible (S) following Yadav et al. [8].

### Dot-blot analysis for disease resistance screening

Dot-blot analysis for screening disease resistance was performed following Sahu et al. [12]. Total genomic DNA was denatured by adding an equal volume of 0.6 M sodium hydroxide and an equal amount of each denatured DNA (~ 1 μg) was spotted onto three Hybond N+ membranes (Amersham Bioscience) embedded in a BIO-DOT dot-blot apparatus (Bio-Rad). The samples were spotted in 96-well formats to prepare three identical arrays. The membranes were hybridized with α^32^P-dCTP labeled DNA A specific AC1 gene sequence, and then neutralized with neutralization buffer (0.5 M Tris-Cl, pH 7.4, 1.5 M NaCl) for 3 min, washed with 2% standard saline citrate (SSC) and cross-linked using UV cross-linker (Stratagene). Radiolabeled probe was made by random primer labeling method by using Megaprime DNA labeling system (Amersham Biosciences) [12].

### Whole genome re-sequencing

Whole genome re-sequencing and further downstream analyses were performed according to Jain et al. [13]. Precisely, genomic DNA from 25 days-old seedlings of resistant cv. ‘UPSM-534’ and susceptible cv. ‘JS-335’ was isolated using Qiagen DNeasy kit (Qiagen) and its quality and quantity was ascertained by Bioanalyzer 2100 (Agilent Technologies). The library preparation was performed using TruSeq DNA PCR-Free HT Sample Preparation Kit, following the manufacturer’s procedure. The libraries were processed for paired-end sequencing using Illumina HiSeq 2000 platform (Illumina Technologies) to generate 100 base long reads and the low-quality reads as well as reads containing adaptor/primer contamination were removed using QC Toolkit v2.3 (http://59.163.192.90:8080/ngsqctoolkit/).

### Read mapping and analysis of SNPs and InDels

The high-quality filtered reads were mapped on to soybean reference genome (*Glycine max* var. Williams 82) using Bowtie v1.0.0 (http://bowtie-bio.sourceforge.net/index.shtml). The recent version of soybean genome assembly (GCA_000004515.1) was retrieved from NCBI (http://www.ncbi.nlm.nih.gov). The resultant mapping data was filtered to identify the reads that were mapped on to only one position of the reference genome. FastQC v0.10.1 (http://www.bioinformatics.babraham.ac.uk/projects/fastqc/) was used for nucleotide base quality filtration. SAMtools v0.1.16 was used to analyse the coverage of reference genome.

The high-quality filtered reads were used for identification of SNPs and InDels using SAMTools v0.1.18 by individually comparing the UPSM-534 and JS-335 genomes with the reference genome. The identified SNPs and InDels were filtered based on default parameters with some minor modifications such as the minimum variant frequency of 90%, average quality of the SNP base of 30 and minimum read depth of 4. Calculation of genomic distribution, frequency and position of DNA polymorphisms were performed for each chromosome. Variant effect analysis of SNPs and InDels, and their positions in the genome was predicted using SnpEff (http://snpeff.sourceforge.net/). Non-synonymous and synonymous changes (non-synonymous substitution is a nucleotide mutation that alters amino acid sequence of a protein whereas synonymous substitution does not alter amino acid sequence) were identified with the effect predictor of SnpEff v3.1h. The distribution of SNPs and InDels on each soybean chromosomes was visualized using Circos [14].

### Annotation of SNPs and InDels

*G*. *max* annotation files (Gmax_189_gene_exons.gff3, Gmax_189_gene.gff3 and Gmax_189_annotation_info.txt) were retrieved from Phytozome (http://www.phytozome.net/) and the data was used to identify the positions of the genes, gene identifiers, functional descriptions and positions of exons, introns and UTRs. Synonymous and non-synonymous substitutions were identified using the procedure described in Kobayashi et al. [15]. Statistical analysis was performed using Microsoft Excel.

### DNA polymorphism detected in known disease resistant QTLs

Disease resistant QTLs reported in Soybase (http://www.soybase.org/) were taken into consideration for the present study. Different classes of resistance (R) genes [CN, genes containing coiled-coil (CC) and nucleotide-binding site (NBS) domain; CNL, genes containing CC, NBS and leucine-rich repeat (LRR) domain; Mlo-like, mildew resistance loci; N, genes containing NBS domain; NL, genes containing NBS and LRR domain; RLK, Receptor-like Kinases; RLK-GNK2, RLK- ginkbilobin-2; RLP, Receptor-like Proteins; RPW8-NL, Resistance to Powdery Mildew8 with NBS and LRR domains; T, genes containing Toll/interleukin-1 receptor-like (TIR) domain; TNL, genes containing TIR-NBS-LRR domains] belonging to disease resistant QTLs were retrieved from PRG database (Plant Resistant gene database; http://prgdb.crg.eu/). The conserved domains in the proteins were predicted by searching against Pfam database using HMMscan v3.0 [16]. Putative functions of the genes were predicted through BLAST search. Blast2GO [17] was performed for functional categorization and gene ontology analysis and the results were visualized by WEGO (Web Gene Ontology Annotation Plot) [18]. MapChart program [19] was used to construct the physical map.

### Expression profiling of disease resistance genes with sequence polymorphisms

Gene Expression data was downloaded from soybean functional genomics database (http://bioinformatics.cau.edu.cn/SFGD/). The differential gene expression data belonging to disease resistant genes were visualized in MultiExperiment Viewer (MeV 4.9.0) software [20,21].

### Validation of SNPs in soybean cultivars

Thirty SNPs were selected (~2 from each chromosomes) and verified for their presence in both the cultivars [UPSM-534 and JS-335] by Sanger sequencing. Approximately 500 bp flanking sequences of the selected SNPs were extracted using in-house perl programming and then flanking forward and reverse primers were designed. The sequences were amplified by PCR using genomic DNA of UPSM-534 and JS-335 along with 10 cultivars. The PCR amplification reactions were performed in a 20 μl reaction volume containing 100 ng of genomic DNA, 1x Taq buffer, 2 mM of MgCl_2_, 0.2 mM dNTP (Promega), 0.5 mM each of the forward and reverse primers and 2 units of Taq polymerase (Biotools). The PCR was performed in an iCycler thermal cycler (Bio-Rad). The PCR profile was: one cycle of 3 min at 94°C, 34 cycles of 30s at 94°C, 45s at 60°C, 60s at 72°C and a final extension of 10 min at 72°C. The PCR products were resolved on 1.2% agarose gel and documented (UVP GelDoc). Amplified PCR fragments were eluted and purified for sequencing.

## Result and Discussion

The present study has direct relevance to the current scenario of identifying prominent breeding strategies for MYMIV resistance. Despite the efforts of soybean breeders, no major breakthrough has been achieved for imparting MYMIV resistance in soybean. Hence, molecular marker-based approaches will offer innovative avenues to breeders, geneticists and researchers to investigate the MYMIV resistance in soybean. Further, this will enable the improvement of horizontal resistances that are polygenetically inherited and for which screening and selection is cumbersome. Whole genome re-sequencing is widely practised for large-scale identification of molecular markers and their utilization in mapping of disease resistant loci. This would serve as a valuable resource for fine mapping the disease resistant loci in crop plants, including soybean. Further, high-throughput DNA sequencing and generation of large-scale expression data will provide the opportunity to explore the genetic and genomic variations at resistance genes/QTLs and pave way for conducting further downstream functional assays to clarify their mechanisms of action.

### Infectivity analysis for MYMIV tolerance in soybean cultivars

**Fig 1:**
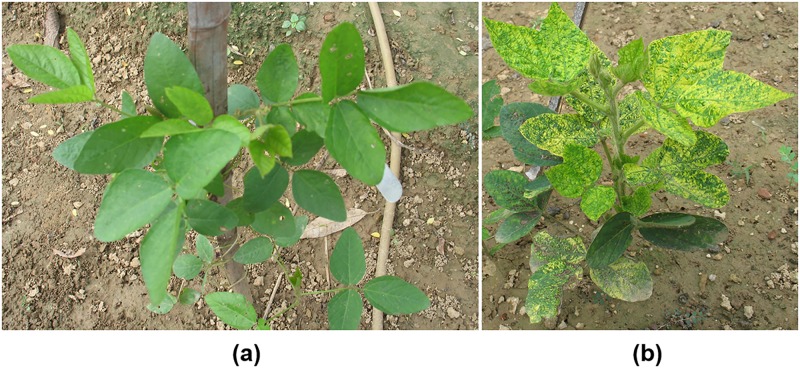
Phenotypic expression of soybean cultivars (a) UPSM-534 [MYMIV tolerant] and (b) JS-335 [MYMIV susceptible] in field conditions.

MYMIV infected soybean seedlings initially showed chlorosis of leaves, which is the characteristic symptom of the disease (Fig 1). The disease progressed with reduced leaf area and finally resulted in stunted growth. The resistant soybean cv. UPSM-534 and susceptible cv. JS-335 along with 10 Indian soybean cultivars were phenotyped as resistant and susceptible based on percentage infectivity at 30 days post inoculation (dpi). Of the 10 cultivars, 5 were found to be resistant (0%), whereas the remaining 5 were susceptible (91–100%) (Table 1). This result was verified by dot-blot of total genomic DNA with AC1 gene sequence used as a probe (Table 1). The dot-blot densities for resistant cultivars ranged from 1.93 to 5.93, whereas for susceptible cultivars, the values ranged from 94.94 to 263.48 (Table 1).

**Table 1:** Infectivity analysis of MYMIV by agroinoculation in soybean seedling of different cultivars.

Cultivars	Dot Blot density value (mean of three replicates)	Symptom severity
JS-335	100	44/45 (99%)
GBPUA&T-1	108.32	45/45 (100%)
GBPUA&T-2	131.48	45/45 (100%)
GBPUA&T-3	168.83	45/45 (100%)
GBPUA&T-4	94.94	41/45 (91%)
GBPUA&T-5	263.48	45/45 (100%)
UPSM-534	5.62	0/45 (0%)
GBPUA&T-6	3.41	0/45 (0%)
GBPUA&T-7	2.52	0/45 (0%)
GBPUA&T-8	3.69	0/45 (0%)
GBPUA&T-9	5.93	0/45 (0%)
GBPUA&T-10	1.93	0/45 (0%)

>90%—Susceptible 0%—Resistant

### Read mapping and coverage of reference soybean genome

A total of 255 million and 234 million potentially paired-end reads were obtained from JS-335 and UPSM-534, respectively. Whole genome re-sequencing was performed with Illumina HiSeq 2000 platform, followed by FASTQ analysis for quality check. Low quality sequences were removed and the high quality reads were mapped on reference genome (*G*. *max* var. Williams 82). Approximately 97% of the reads were mapped to the reference genome with a coverage of 95% in both the cultivars (Table 2). Nearly, 95% of reads were properly paired with reference genome during mapping process (S1 Table). Of these, ~1.5 million reads mapped to more than one chromosome in the soybean genome, whereas ~2.5 million reads were uniquely mapped on the reference genome (Table 2).

**Table 2:** Statistics for high quality reads derived from JS-335 and UPSM-534 which were mapped onto the reference genome of soybean.

Mapping Statistics	JS-335	UPSM-534
**Total Reads**	255055818	234177514
**Mapped reads**	249394439	228850264
**Properly paired**	243055468	222686808
**Itself and mate mapped**	246733728	226319760
**Singletons**	2660711	2530504
**Unmapped reads**	5661379	5327250

### Identification and characterization of SNPs and InDels

**Fig 2:**
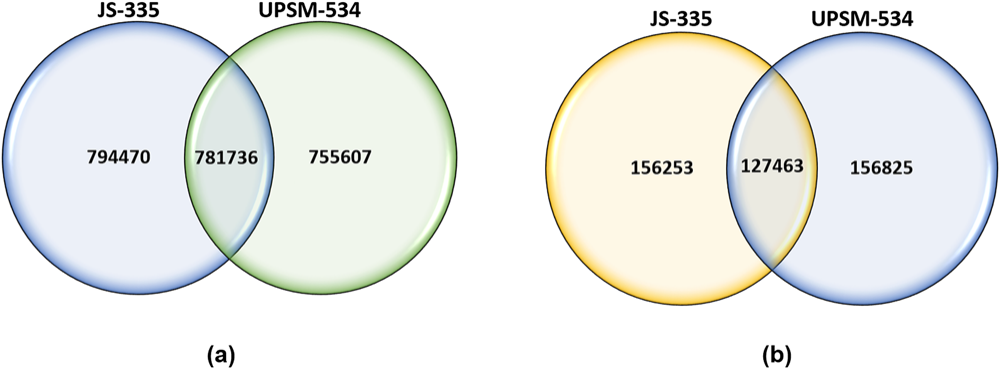
Comparison of (a) SNP and (b) InDel distributions between both the cultivars (JS-335 and UPSM-534).

A total of 1524431 and 1559556 SNPs as well as 280900 and 281958 InDels were identified in JS-335 and UPSM-534, respectively, by their individual comparison with reference soybean genome. These SNPs and InDels were filtered at minimum read depth. The read depth of SNPs and InDels varied from a minimum of 5 to more than 100. The overall density of SNP was approximately one change in every 600 bases, and the density of InDels was nearly one change in every 3000 bases. In JS-335, 1576206 SNPs and 283716 InDels were identified, whereas in UPSM-534, 1537343 SNPs and 284288 InDels were found. Noteworthy, 781736 SNPs were identified to be common in both the cultivars as compared to reference soybean genome. This comparative analysis has also revealed the presence of 794470 and 755607 unique SNPs in JS-335 and UPSM-534, respectively (Fig 2).

**Fig 3:**
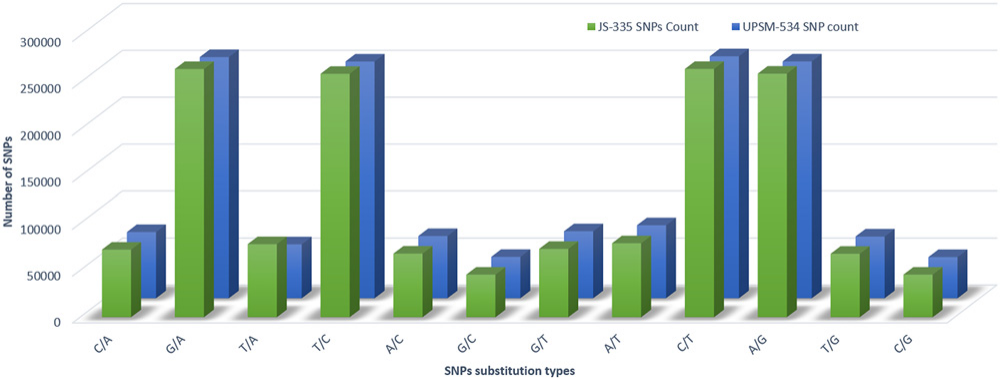
Frequency of different nucleotides substitution types in the identified SNPs from JS-335 and UPSM-534.

**Fig 4:**
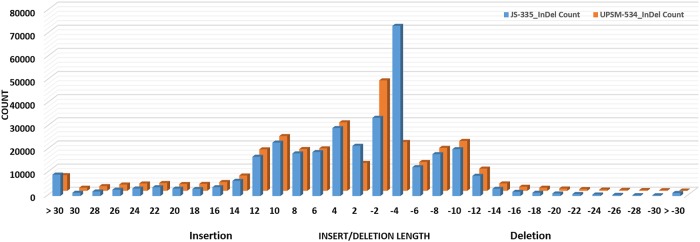
Frequency of length distribution of InDels JS-335 and UPSM-534.

The numbers of transition (Ts) and transversion (Tv) type of SNPs were 1042619 and 527093, respectively, with a Ts/Tv ratio of 1.9781 for JS-335 (Fig 3). Similarly, the numbers of Ts and Tv were 1017711 and 519011, respectively, with a Ts/Tv ratio of 1.9609 for UPSM-534. The length of deletions and insertions in InDels in both the cultivars ranged from 1 to 37 bp and 1 to 65 bp, respectively (Fig 4).

### Genomic distribution of SNPs and InDels across soybean chromosomes

**Fig 5:**
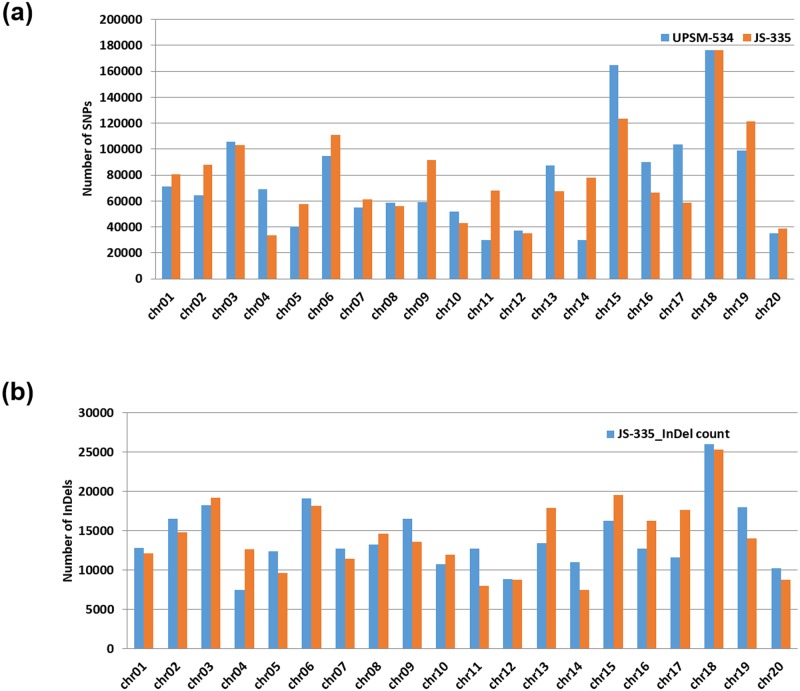
Number and distribution of (a) SNPs and (b) InDels detected on the soybean chromosomes in both the cultivars (JS-335 and UPSM-534).

*Glycine max* var. Williams 82 genome with 950068807 bases contained in twenty chromosomes was used as reference. The distribution of DNA polymorphisms detected between JS-335 and UPSM-534 was analysed with the reference genome. The frequency of SNPs and InDels identified in both the cultivars varied across twenty chromosomes (Fig 5). Maximum number of SNPs and InDels was detected in chromosome 18 and minimum number in chromosome 12 for both the cultivar JS-335 and UPSM-534 (Fig 5). Overall, the frequency of SNPs was higher in JS-335 in comparison to UPSM-534 whereas higher number of InDels was detected in UPSM-534.

**Fig 6:**
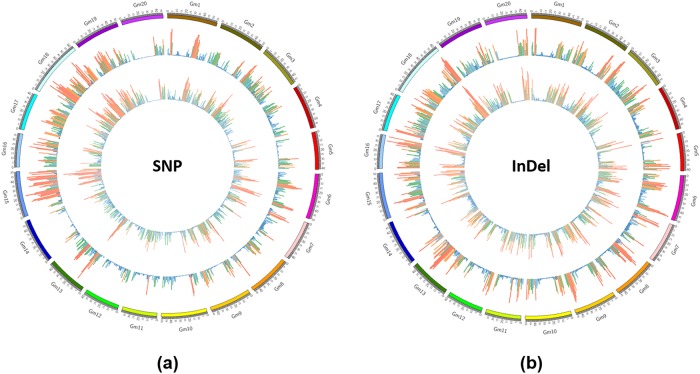
Density distribution of (a) SNP and (b) InDel in all the 20 chromosomes of soybean. JS-335 (outer circle) and UPSM-534 (inner circle).

An uneven distribution of SNPs and InDels across short arm to long arm of soybean chromosomes was observed. An average of 1916.12 and 1584.23 SNPs per Mb was detected in JS-335 and UPSM-534, respectively. Similarly, an average of 390.35 and 376.29 InDels per Mb was detected in JS-335 and UPSM-534, respectively. Higher SNP density was observed in chromosome 18 of both JS-335 and UPSM-534 with 2817.27 SNPs per Mb and 2822.78 SNPs per Mb, respectively, whereas lower SNP density was found in chromosome 4 of JS-335 (672.52 SNPs per Mb) and chromosome 14 of UPSM-534 (606.68 SNPs per Mb). Similarly, higher InDel density was observed in chromosome 18 of JS-335 (441.89 InDels per Mb) and chromosome 16 of UPSM-534 (447.79 SNPs per Mb), whereas lower InDels density was detected in chromosome 4 of JS-335 (158.28 InDels per Mb) and chromosome 14 of UPSM-534 (157.58 InDels per Mb) (Fig 6). Further, the study identified unanimous distribution of SNPs and InDels in chromosome 18 and 19 of both the cultivars. Chromosomes 2, 3, 7 and 9 of JS-335 encompassed higher SNPs within 10 Mb, whereas chromosomes 2, 3, 4, 9 and 12 of UPSM-534 higher SNPs within 10 Mb. Similarly, uneven distribution of InDels within the chromosomes was also evidenced (Fig 6).

### Annotation of SNPs and InDels

**Fig 7:**
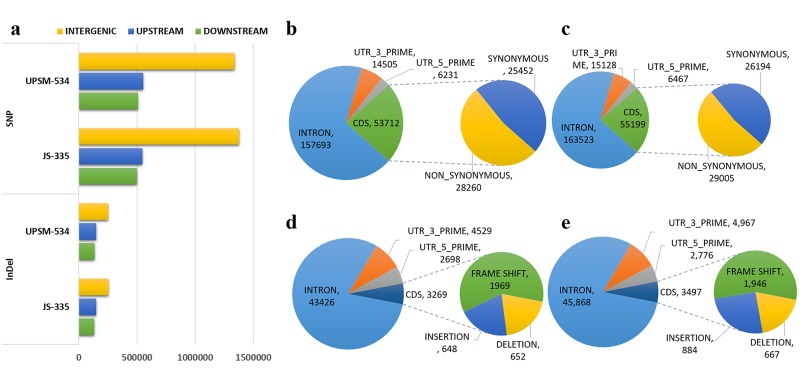
Annotation of single-nucleotide polymorphisms (SNPs) and InDels. **(a)** Distribution of SNPs and Indels in intergenic, upstream and downstream region. **(b, c)** Distribution of SNPs in different genic regions for JS-335 and UPSM-534. **(d, e)** Distribution of InDels in genic regions for JS-335 and UPSM-534. The number of synonymous and non-synonymous SNPs detected within the CDS region has also been shown.

The annotation of soybean reference genome was used to reveal the distribution of SNPs and InDels within various genomic regions such as intergenic and intragenic. Approximately 50% of SNPs were identified in intergenic regions in both the cultivars. Overall, a similar distribution pattern of SNPs and InDels was observed in the entire chromosome for both the cultivars. Only 10–12% of total SNPs were detected in genic regions, whereas upstream (promoter) and downstream regulatory regions had significant number of SNPs (18–22%). Within the genic region, more than 6% of SNPs were present in the introns (Fig 7). The 3′ UTR and 5′ UTR regions also showed the presence of SNPs (0.5–1.0%). Similarly, ~42% of InDels were identified in intergenic regions in both the cultivars. Only 0.5% of total InDels were present in exonic regions, whereas upstream (promoter) and downstream regulatory regions contained ~20% InDels. Within the genic region, nearly 7% of InDels were present in the introns (Fig 7). Similar to SNPs, the presence of InDels (0.3–0.8%) was also observed in 3′ UTRs and 5′ UTRs (Fig 7). The abundance of InDels in the upstream and downstream regulatory regions of genes is expected because of low sequence conservation and reduced purifying selection pressure in these non-coding regulatory regions as compared to coding regions [13].

### Effect of SNPs on amino acid substitution

Amino acid substitution was analysed to investigate the effect of SNPs in coding sequences. In JS-335, 28267 and 25533 SNPs showed non-synonymous and synonymous type modifications, respectively. Similarly, 29012 non-synonymous and 26195 synonymous changes were observed in UPSM-534. The ratio of non-synonymous to synonymous SNPs was about 1.11 for JS-335 and 1.10 for UPSM-534 (Fig 7). Functional annotation of these genes revealed putative roles of respective proteins in plant defence machinery. The genes encoding for various developmental and regulatory pathways including pentatricopeptide repeat, leucine-rich repeat and protein kinase family related genes were identified (S2–S4 Tables; S1 and S2 Figs).

### DNA polymorphism in disease resistant QTLs and differential expression of resistance genes

Functional annotation of the genes showing SNP variations revealed the participation of these genes in defense response. A total of 1514 SNPs were detected in 564 defence response-related genes in both the soybean cultivars (S2 and S3 Tables) and distributed across all the chromosomes. These genes were further searched for domain organizations in respective genes. Defence response proteins were found to contain nucleotide binding site and LRR domain including, GNK2, MLO, NBS, RPW, receptor like kinase, Ser-Thr Kinase and TIR domains, which are involved in host-pathogen interaction and defence response (S4 Table). Of the 1514 SNPs, 829 non-synonymous and 671 synonymous SNPs were detected in 266 and 286 defence-related genes, respectively. QTLs coinciding with the defence related genes were further analysed for the differential expression using publicly available expression database. The results showed the diverse spatial- and temporal-expression patterns of these genes (S5 Table; S3 and S4 Figs).

Although the use of next-generation sequencing and high-throughput analysis platforms in re-sequencing of soybean cultivars and identification of QTLs related to biotic stress tolerance have been demonstrated [22–27], no attempt has been made to re-sequence soybean cultivars with contrasting tolerance behaviour to MYMIV and identify the putative disease-resistance QTLs. Lam et al. [24] re-sequenced 17 wild and 14 cultivated soybean genomes to identify the pattern of genetic diversity and selection, whereas Xu et al. [25] re-sequenced 246 recombinant inbred line populations to pinpoint the QTLs for root-knot nematode resistance in soybean. Similarly, Chung et al. [26] re-sequenced 10 cultivated and 6 wild soybean accessions to reveal their population structure and domestication. Li et al. [27] re-sequenced 25 diverse soybean accessions including, 8 wild soybeans, 8 landraces and 9 modern elite cultivars to analyze the molecular footprints of domestication.

### Validation of SNPs in soybean cultivars

**Fig 8:**
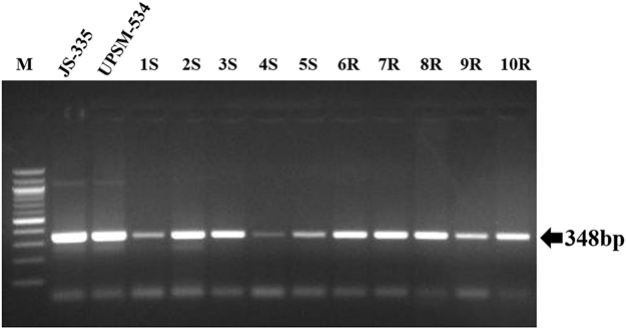
PCR amplification of 12 genomic DNA samples using 18–1861613 primer. M, Marker; JS-335 (susceptible); UPSM-534 (resistant); 1S - 5S, susceptible cultivars; 6R - 10R; resistant cultivars.

**Fig 9:**
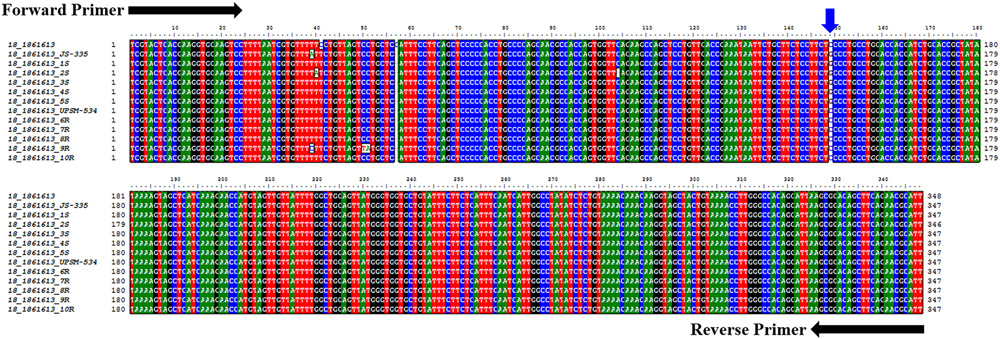
Multiple alignment of sequences obtained from 18–1861613 primer in 12 soybean cultivars using *Glycine max* var. ‘Williams 82’ genome as reference. SNP position is indicated with blue down arrow.

**Fig 10:**
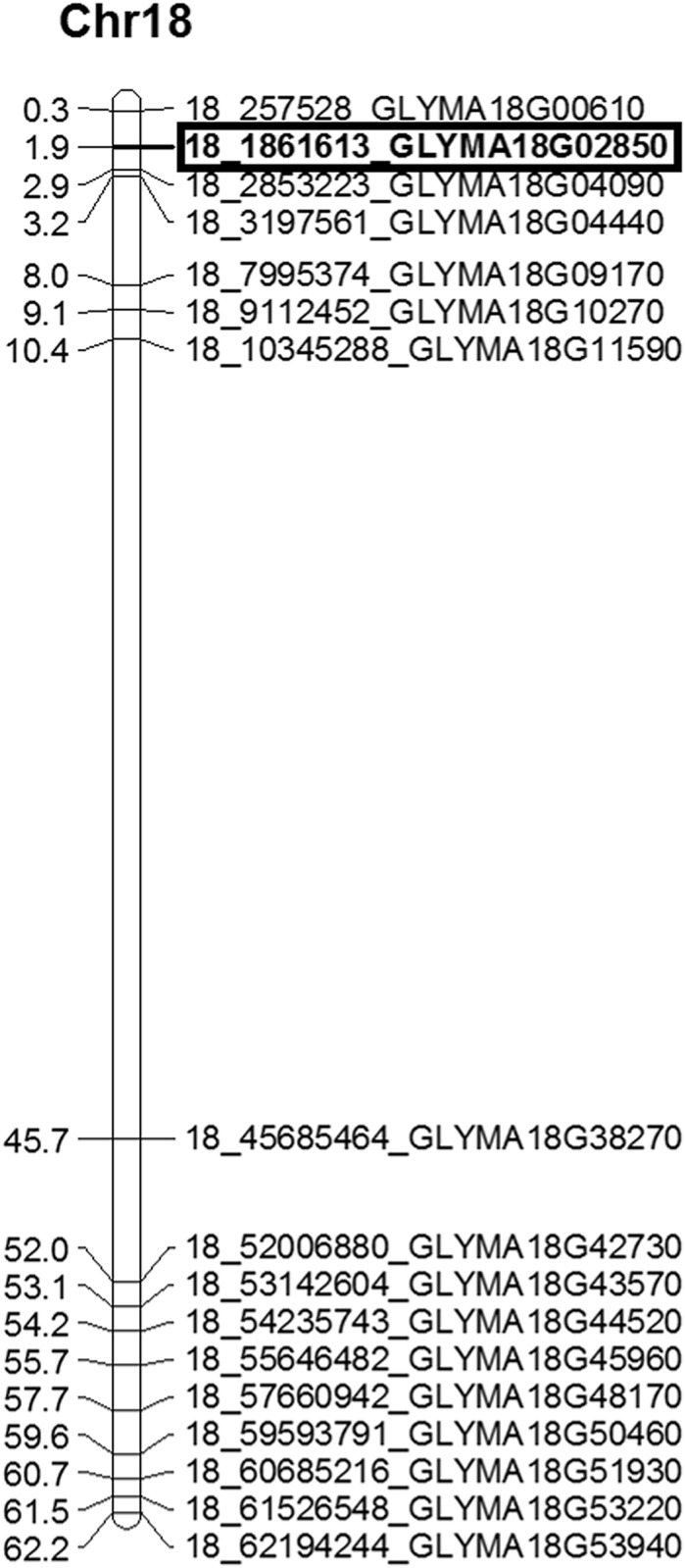
Physical map showing representative SNPs and the LRR-RP linked SNP 18–1861613 highlighted in a box. The numbers at the left indicate the physical position of respective SNPs in megabase and the SNP IDs are shown in the right. The complete physical map of 1514 SNPs detected in 564 defence response-related genes is shown in S3 Fig.

A total of 30 primers targeting 76 SNPs were selected for validation based on their presence in genes which were associated with disease resistance-related QTLs (S6 Table). The SNPs were amplified by flanking primers in 12 soybean cultivars (Fig 8). The amplicons were re-sequenced and found that the sequences showed perfect similarity with the reference genomes with variation at single nucleotide. A frequency of 5.06 SNPs/Mb was observed in the re-sequenced 12 Indian soybean cultivars. Of note, a non-synonymous SNP in chromosome 18 (18–1861613) was observed to be associated with MYMIV-resistance at the 149 base-pair (a G/C transversion) in the *LEUCINE-RICH REPEAT RECEPTOR-LIKE PROTEIN KINASE* (*LRR-RP*) gene (Glyma18g02850) (Fig 9). The G to C transversion causes a codon change of CCC to GCC, resulting in a mis-sense non-synonymous substitution of proline to alanine. Noteworthy, this SNP is not present in the functional domain of *LRR-RP* gene. The physical location of the *LRR-RP* gene is shown in Fig 10. LRR-RPs are a class of nucleotide binding site—leucine-rich repeat (NBS-LRR) playing crucial roles in resistance to a broad-spectrum of phytopathogens [28,29]. The SNP identified in the present study would serve as an important source for the improvement of disease resistance in soybean through either molecular breeding or transgene-based approaches. Further, development of CAPS/allele-specific marker for this SNP is in progress. Precisely, these types of synonymous and non-synonymous changes may affect the expression, structural and functional changes in gene product towards the alteration in various developmental phenomena, particularly the tolerance to various diseases.

## Conclusions

In the present study, SNPs and InDels were generated in large-scale by re-sequencing two soybean cultivars with contrasting tolerance characteristics to MYMIV infection. In addition to the usefulness of these resources in genomics-assisted breeding for MYMIV resistance, the present study will facilitate the dissection of complex traits through construction of high-density linkage map and fine mapping. Further, these markers would be useful for high-density SNP array designing and high-throughput genotyping for variety identification, gene tagging, molecular mapping of genes/QTLs, construction of linkage map, association mapping and marker-assisted selection. Of note, the SNP identified in chromosome 18 (18–1861613) with G/C transversion at 149 bp could be used in marker-assisted breeding for MYMIV resistance after large-scale validation.

## Supporting Information

S1 Fig Gene Ontology distribution for genes, which showed SNPs by comparing with reference genome. (TIF)

S2 Fig Gene Ontology distribution for genes, which showed InDels by comparing with reference genome. (TIF)

S3 Fig Physical location of 1514 SNPs detected in 564 defence response-related genes on soybean chromosomes. The SNP 18–1861613 is highlighted in red. Non-synonymous SNPs are indicated in bold, synonymous SNPs are underlined.
(TIF)

S4 Fig Expression of R genes, which showed SNP and InDels. (TIF)

S1 Table The distribution of reads per chromosomes in both the cultivars. (XLS)

S2 Table Non-synonymous coding changes due to SNP in R genes. (XLSX)

S3 Table Synonymous coding changes due to SNP in R genes. (XLSX)

S4 Table Frameshift variation/insertion/deletion due to InDels in R genes. (XLSX)

S5 Table Expression of R genes, which showed SNP and InDels. (XLSX)

S6 Table List of the primers for SNP validation in both the cultivars. (XLSX)
