# Supplementary figures and images for: Genome-Wide SNP Identification and Characterization in Two Soybean Cultivars with Contrasting Mungbean Yellow Mosaic India Virus Disease Resistance Traits
Source: PLoS One. 2015 Apr 13;10(4):e0123897. doi: 10.1371/journal.pone.0123897 (PMC4395324; doi:10.1371/journal.pone.0123897)

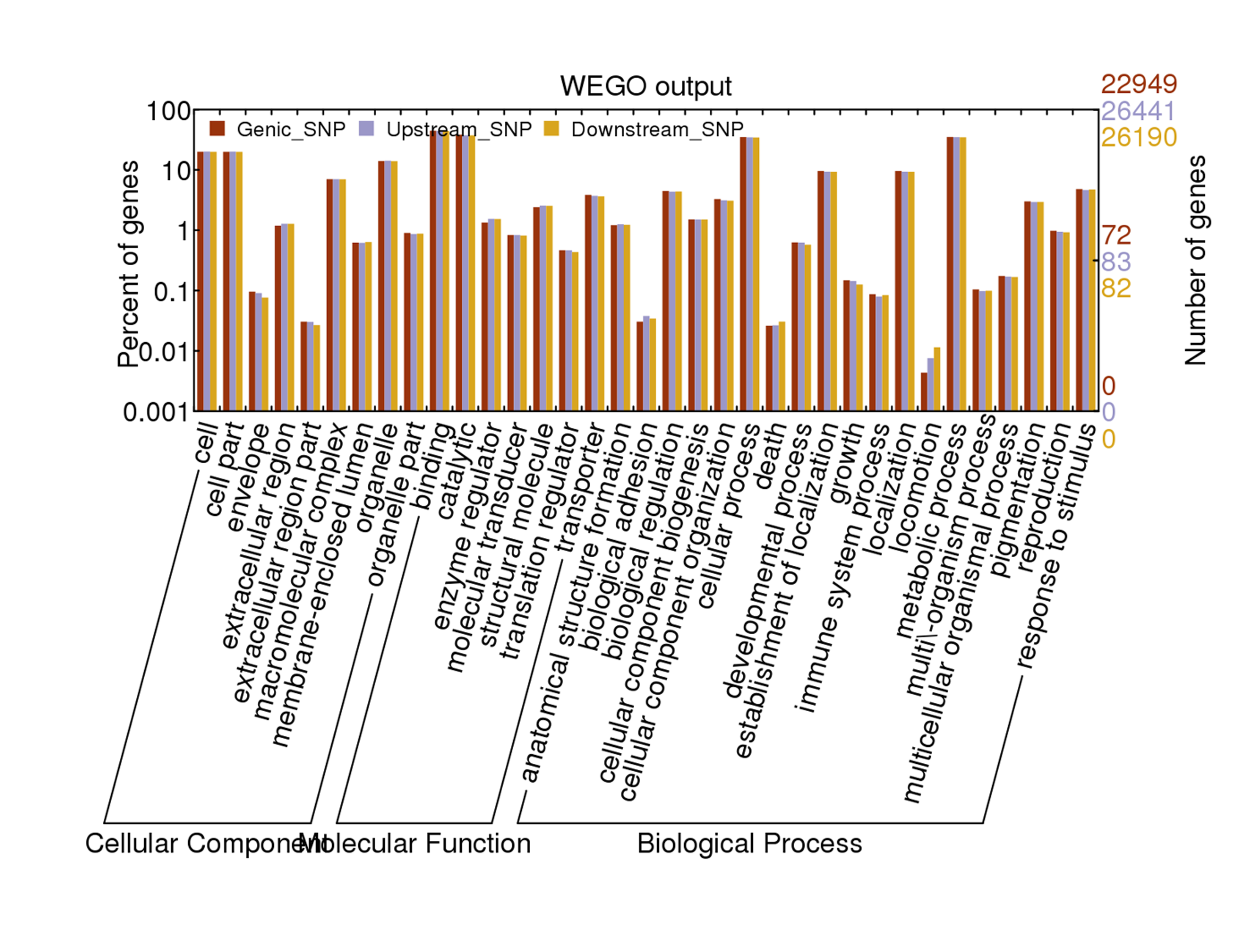

Supplement: S1 Fig — (TIF) [file pone.0123897.s001.tif]

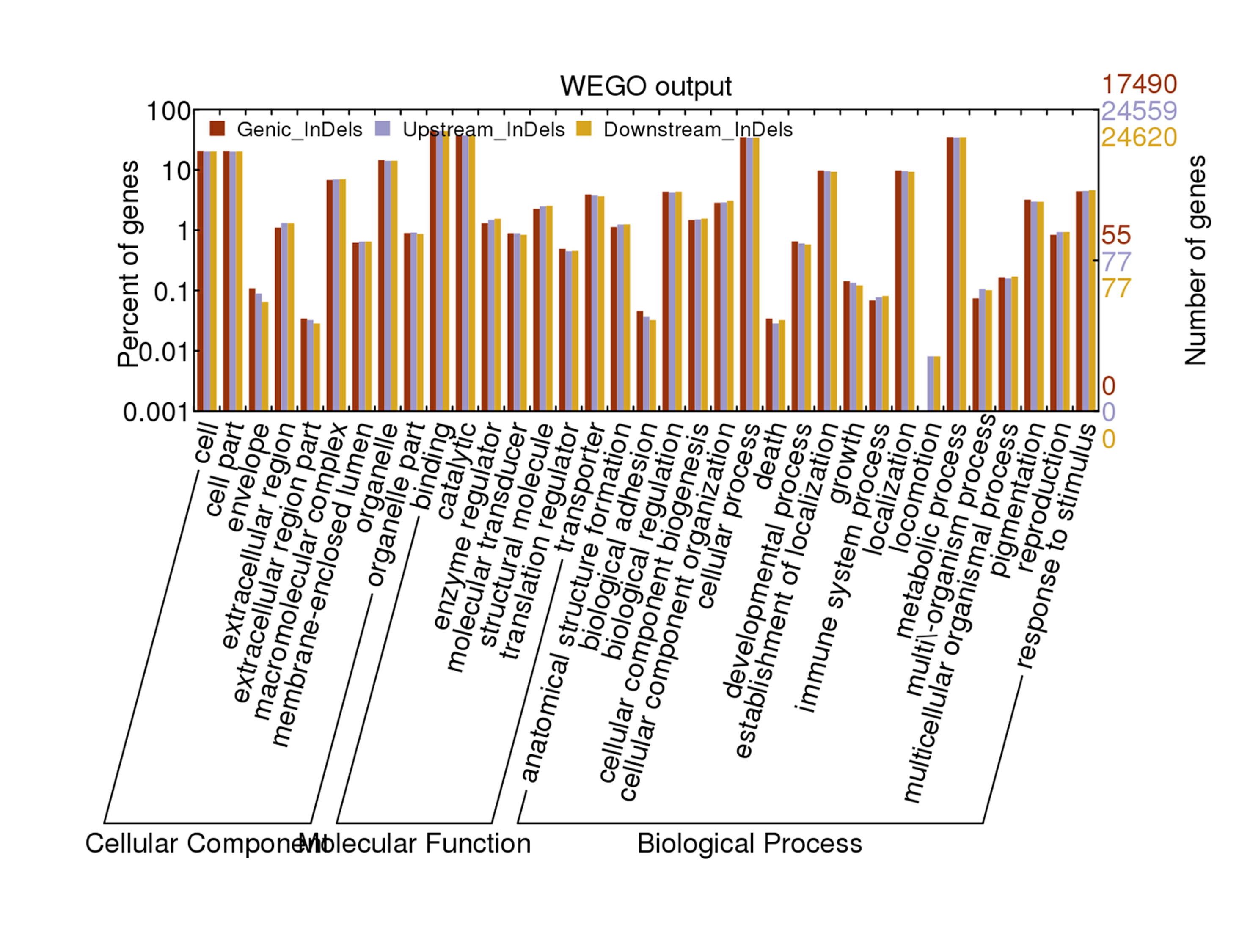

Supplement: S2 Fig — (TIF) [file pone.0123897.s002.tif]

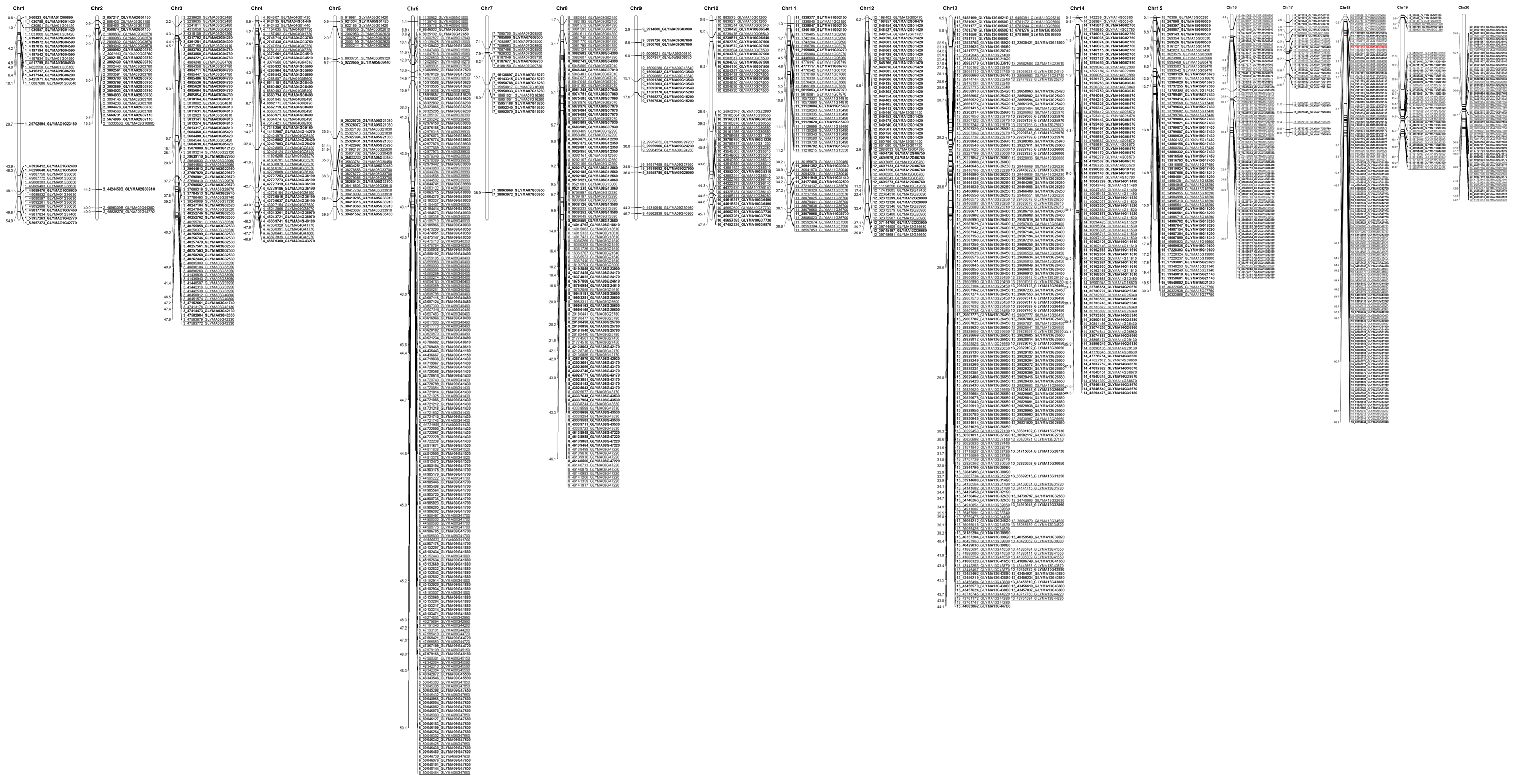

Supplement: S3 Fig — The SNP 18–1861613 is highlighted in red. Non-synonymous SNPs are indicated in bold, synonymous SNPs are underlined. (TIF) [file pone.0123897.s003.tif]

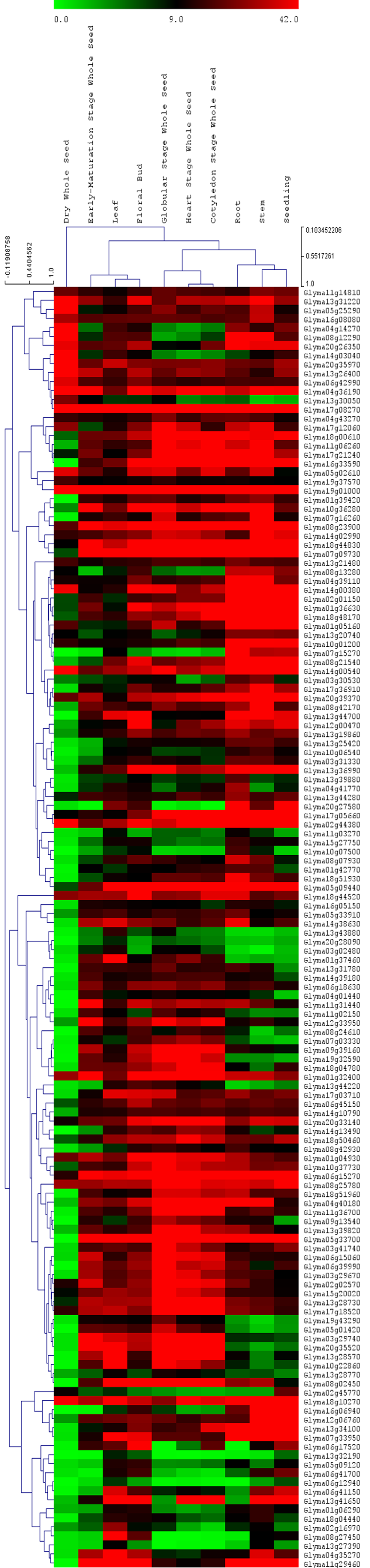

Supplement: S4 Fig — (TIF) [file pone.0123897.s004.tif]
